# The oasis effect and summer temperature rise in arid regions - case study in Tarim Basin

**DOI:** 10.1038/srep35418

**Published:** 2016-10-14

**Authors:** Xingming Hao, Weihong Li, Haijun Deng

**Affiliations:** 1 State Key Laboratory of Desert & Oasis Ecology, Xinjiang Institute of Ecology & Geography, Chinese Academy of Sciences, Urumqi, China

## Abstract

This study revealed the influence of the oasis effect on summer temperatures based on MODIS Land Surface Temperature (LST) and meteorological data. The results showed that the oasis effect occurs primarily in the summer. For a single oasis, the maximum oasis cold island intensity based on LST (*OCI*_LST_) was 3.82 °C and the minimum value was 2.32 °C. In terms of the annual change in *OCI*_LST_, the mean value of all oases ranged from 2.47 °C to 3.56 °C from 2001 to 2013. Net radiation (*R*_n_) can be used as a key predictor of *OCI*_LST_ and *OCI*_temperature_ (OCI based on air temperature). On this basis, we reconstructed a long time series (1961–2014) of *OCI*_temperature_ and *T*_base_(air temperature without the disturbance of oasis effect). Our results indicated that the reason for the increase in the observed temperatures was the significant decrease in the *OCI*_temperature_ over the past 50 years. In arid regions, the data recorded in weather stations not only underestimated the mean temperature of the entire study area but also overestimated the increasing trend of the temperature. These discrepancies are due to the limitations in the spatial distribution of weather stations and the disturbance caused by the oasis effect.

An oasis is a type of medium-sized or small-sized non-zonal landscape that occurs in dry climates and is supported by natural or artificial rivers in deserts^1^,^2^. Oases are characterized by a comparatively high primary productivity and the presence of mesophytic or xero-mesophytic plants as the dominant vegetation^3^,^4^. Oases perform an important role in the arid region of Northwest China; although their total area only accounts for 4% to 5% of the total land area, they carry 90% of the population and supply 95% of the total economic output^5^,^6^.

In arid regions, because of high temperature and drought, the oasis effect, which was defined as “the vegetation cooling effect due to extensive evapotranspiration in oasis compare to the surroundings, is particularly prominent. The oasis effect was first confirmed by a numerical model of the planetary boundary layer^7^. Several subsequent studies have indicated that the oasis effect is a ubiquitous phenomenon in arid regions, including Northwest china and Southern Israel^2^,^8^,^9^,^10^,^11^,^12^. Furthermore, the oasis effect improves the regional microclimate^13^; in particular, it significantly decreases the temperature in the oasis area. Studies showed that summer air temperature of oasis can be cooler 2–7 °C than their surroundings in desert environments^14^,^15^.

Although the oasis effect is clear, the observed temperature still shows an increasing trend in arid regions^16^,^17^,^18^. Several studies suggest that, during the past 60 years, the temperature rise was 0.32–0.36 °C/decade and 0.1–0.3 °C/decade in the arid regions of Northwest China and in the Tarim Basin, respectively^19^,^20^. Global warming is mainly caused by the emissions of greenhouse gases, including carbon dioxide, methane, nitrous oxide and various halogen-containing compounds^21^. On a regional scale, the temperature rise in the arid region of Northwest China, which includes the Tarim Basin, can generally be attributed to changes in atmospheric circulation, such as El Niño-Southern Oscillation (ENSO)^20^, the Siberian High^22^, the North Atlantic Oscillation (NAO) or the Arctic Oscillation (AO)^23^,^24^.

However, few studies have acknowledged that the oasis effect also has an important influence on the temperature background level and the temperature changing trend in arid areas. Especially in arid region, most of the weather stations are located in oasis (close to the town) due to the limitation of the environmental condition and the working condition. Therefore, the observed temperature not only can’t represent the temperature background of whole region, but also can’t eliminate the disturbance of oasis cold island on temperature. For example, in the Tarim Basin, there are a total of 43 weather stations, but only one station is located in the desert and four stations are distributed in the mountainous area (altitude higher than 1500 m), and the other 39 stations are distributed in oases, which account for only 11.5% of the plains area. Unfortunately, these factors have been ignored by most researchers when study the temperature changing in this area.

In order to truly reflect the changing trend of temperature in arid area, such as in Tarim Basin, we need to evaluate the *OCI* and reconstruct the temperature time series which without the disturbance of oasis cold island effect.

Thus, the present study take Tarim Basin as the study area and assessed the oasis effect and its potential influence through ArcGIS spatial analysis and statistical methods based on the MODIS LST and meteorological data. The aims of this study are to 1) reveal the characteristics and controlling factors of the oasis effect; 2) construct a long time series of *OCI*_temperature_ (oasis cold island index based on air temperature) and calculate the summer air temperature without the oasis effect; and 3) evaluate the impact of the oasis effect on the summer temperature, especially its influence on the temperature changing trend.

## Results

### The oasis effect in the Tarim Basin

**Figure 1:**
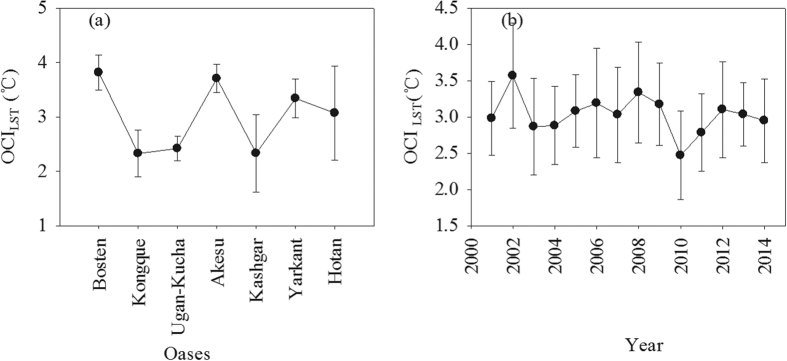
The multi-year average OCI_LST_ (oasis cold island intensity based on LST data) changing trend in the seven main oases during 2001 to 2013 (**a**), and the average OCI_LST_ of all the oases (**b**) in the Tarim Basin from 2001–2013. The OCI_LST_ was calculated based on the LST of each oasis and desert buffer zone. The error line in panel (a,b) present the standard deviation of inter-annual OCI_LST_ in each oasis and spatial variation of all oases’ OCI_LST_ in each year, respectively. All *OCI* was calculated in summer season.

The oasis effect was significant during the summer and exhibited a clear spatial and temporal variance. We calculated all OCI was in summer season. The results indicate that, for a single oasis, the maximum value of the multi-year average *OCI*_LST_ was 3.82 °C, which occurred in the Bosten oases, whereas the minimum *OCI*_LST_ was 2.32 °C, which occurred in the Kongque oasis from 2001 to 2013. In other oases, the multi-year average *OCI*_LST_ from high to low was 3.71 (Akesu oasis), 3.34 (Yarkant oasis), 3.07 (Hotan oasis), 2.42 (Ugan-Kucha oasis) and 2.33 °C (Kashgar oasis) (Fig. 1a). An inter-annual fluctuation of the *OCI*_LST_ was also revealed. The standard deviation analysis of the *OCI*_LST_ in a single oasis showed that, from 2001 to 2013, the inter-annual change of *OCI*_LST_ was the most intense in the hotan oasis, followed by the Kashgar, Kongque, Yarkant, Bosten, Akesu and Ugan-Kucha oases; the standard deviation was 0.86, 0.71, 0.42, 0.35, 0.32, 0.26 and 0.23, respectively (Fig. 1a). For the entire basin, the annual average *OCI*_LST_ of all the oases ranged from 2.47 °C to 3.56 °C (Fig. 1b).

### The control factor of OCI

**Figure 2:**
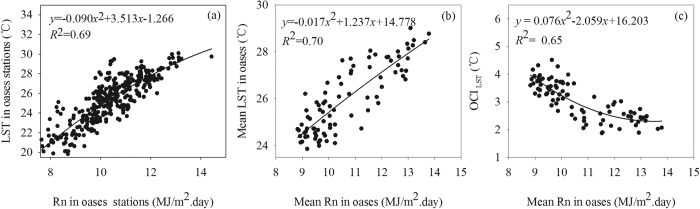
The scatter plot between the LST, *OCI*_LST_ (OCI calculated based on the LST data) and *R*_n_ in oases. The panel (a) is the scatter plot between LST and *R*_n_ of weather stations in all oases during 2001 to 2013; the panel (b) is the scatter plot between the mean LST and *R*_n_ in each oasis of the seven oases region from 2001 to 2013. Panel (c) is the scatter plot between the mean *OCI*_LST_ and *R*_n_ in each oasis of the seven oases region during 2001 to 2013. The *R*_n_ data were calculated from the observed meteorological data from weather stations, and the LST data were extracted from MODIS LST dataset based on the locations of weather stations.

In general, the stronger evapotranspiration in oases area often lead to the lower temperature in oases area. The *R*_n_ is the crucial factor of surface energy balance, and it brings an important influence on LST and cold island intensity of oases. The Fig. 2a showed that the LST and *R*_n_, which was calculated based on weather stations data in the oases area, had a positive correlation and the increase in *R*_n_ usually lead to the increase in LST based on the surface energy balance. On the basis of the meteorological station data, the paper further statist iced the average LST and *R*_n_ in each oasis area (Fig. 2b). The results showed that in the oasis areas, the mean LST and *R*_n_ also indicated a positive correlation and could be fitted by a polynomial regression formula. Similar to the changing trend in every station, the increase in mean *R*_n_ usually lead to a obviously increase in mean LST in the entire oases area. Because the OCI was the difference in LST between desert and oases, the *OCI*_LST_ had a significant and positive correlation with *R*_n_ in the oases. Figure 2c shows that the *OCI*_LST_ can also be fitted by a polynomial regression formula, which indicates that the *R*_n_ can be used as a key predictor of the *OCI*_LST_ in the oases based on the data from seven oases from 2001 to 2013.

### *OCI*_temperature_ and *T*_base_ sequence reconstruction

**Figure 3:**
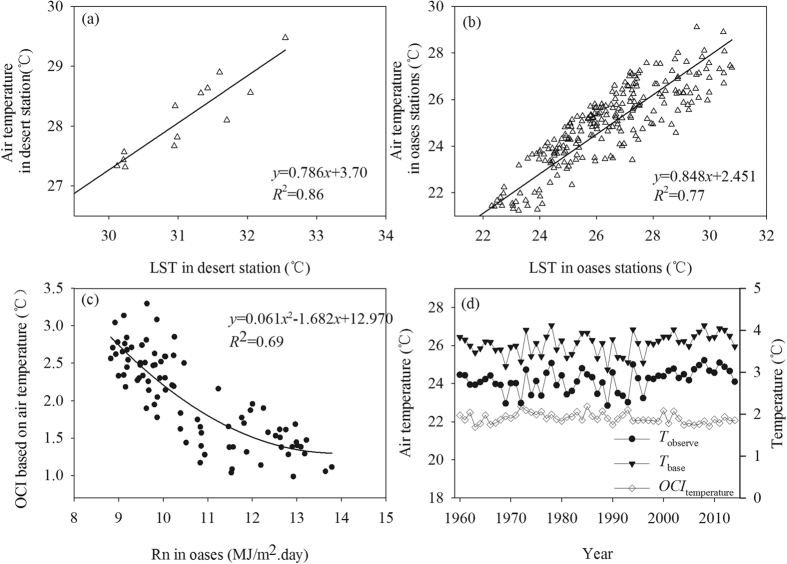
Reconstructed summer *T*_base_ time series and the observed air temperature sequence from 1960 to 2014 (**d**) by using the correlation relationship between *OCI*_temperature_ and *R*n in oases during 2001 to 2013 (**c**). The *OCI*_temperature_ was calculated with the average temperature in the desert (**a**) and the oasis zones (**b**), which was fitted by LST from 2001 to 2013. Panel (a) shows the air temperature data collected from the Tazhong station (the only desert station) and the LST data extracted from the Tazhong station point by using ArcGIS 10. In panel (b), all the temperature data were collected from weather stations in the oases, and the LST data were extracted from these station points using ArcGIS 10.

To reveal the impacts of the oasis effect on the air temperature for long time series, we firstly reconstructed the sequence of *OCI*_temperature_ (oasis cold island intensity calculated based on air temperature). Based on the observed air temperature data and the MODIS LST data of every weather station in the summers from 2001 to 2013, we established a regression equation between air temperature and LST in the oasis and desert areas (Fig. 3a,b). Then, the *OCI*_temperature_ of different oases was calculated based on the mean air temperature in the different oases and desert buffer zone, which was simulated using the fitting equation in Fig. 3a and Fig. 3b. The results of Fig. 3c suggest that the *OCI*_temperature_ can be well fitted by *R*_n_ in the oases. Thus, we obtained the long sequence of *OCI*_temperature_ by the above fitting equation based on the time series data of *R*_n_ from 1961 to 2014.

Because the oasis effect is an objective reality, and the observed air temperature in oasis area was under the influence of oasis effect. Thus, this study set the mean background air temperature without the influence of oasis effect as *T*_base_ and the observed air temperature as *T*_observe_. Furthermore, the relation between *T*_base_ and *T*_observe_ is as follows:


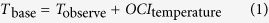


Based on equation (1), the mean *T*_base_ of all the oases was reconstructed from 1961 to 2014 (Fig. 3d). The results showed that the mean *T*_base_ of all the oases was 1.87, 2.00, 1.96, 1.88 and 1.85 °C higher than the *T*_observe_ in the 1960s, 1970s, 1980s, 1990s and since 2000, respectively.

The Mann-Kendall trend analysis indicated that the *T*_observe_ extremely significant increased during 1961–2014 (Test Z = 2.94, 99% confidence level), and the average temperature rise was 0.15 °C/decade. The *OCI*_temperature_ significantly decreased during the same period (Tset Z = −2.48, 95% confidence level), with an average decrease of 0.06 °C/decade. As to the *T*_base,_ it only showed a slight increase changing trend over the past 50 years(Tset Z = 1.94, 90% confidence level), and the average temperature rise only was 0.10 °C/decade, which far less than the increase trend of *T*_observe_. These results suggest that the significant increasing trend of *T*_observe_ could be mainly attributed to the decreasing changing trend of the *OCI*_temperature_ from 1961 to 2014. In other words, the contribution rate of the *OCI*_temperature_ decrease to the *T*_observe_ increase was much higher than that of the *T*_base_ increase during the last 50 years.

## Discussion

### The oasis effect and its influencing factors

The oasis effect was the result of higher water consumption in the oasis areas^3^ and the evapotranspiration from the oasis surface, which cools the oasis (Chu *et al.*^9^). The evapotranspiration of arid regions is the highest in the summer; thus, the OCI is also the highest in summer^15^. Our study showed that for a single oasis, the maximum *OCI*_LST_ and *OCI*_temperature_ were 4.52 °C and 2.88 °C in summer, respectively. Several factors can affect the oasis effect, such as the mesoscale and secondary circulation^5^,^9^,^25^, background winds^7^, surface conditions^26^,^27^ and the landscape or type metrics of the Land-Use and Land-Cover Change (LUCC)^15^.

Although several factors can affect the oasis effect, the *R*_n_ was the driving force of the OCI based on our results. Indeed, the oasis effect was the result of the higher evapotranspiration in the oases than in the surrounding desert environments. Therefore, the evapotranspiration between the oasis and the desert should be a crucial factor in determining the OCI. Therefore, the OCI can be expressed as a function of the difference between evapotranspiration (*ET*) in the desert and oases:


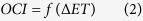


Based on the surface energy balance equation, the *ET* is given as follows:


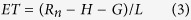


where *R*_n_ is the net radiation; *H* and *G* are the sensible heat flux and soil heat flux, respectively; and *L* is the *latent heat of* vaporization of water, which is a constant. *H* and *G* also can be expressed as a function of temperature^28^,^29^. Furthermore, the temperature can be fitted by *R*_n_ based on our results (Figs 2 and 3). Therefore, *R*_n_ can be used as the key predictive factor of the OCI. In addition, based on the surface energy balance equation, a change in *R*_n_ will cause a change in the latent heat flux (evapotranspiration) and sensible heat flux (air temperature or LST)^28^. In the desert area, the change in *R*_n_ can directly affect the temperature because the evapotranspiration in the desert is limited by the water supply^30^,^31^. In the oasis area, because of the adequate water supply^32^,^33^, the change in *R*_n_ not only influences the temperature but also affects the evapotranspiration. This relation implies that in both desert and oasis areas, the change of *R*_n_ will cause the corresponding change in temperature, which finally determines the OCI (Fig. 3c).

In most case, the rise of temperature will lead to the increase of evapotranspiration, which will finally enhance the oasis effect. However, our research showed an interesting result that the *OCI*_temperature_ showed a decreasing trend with the increasing of air temperature in oasis area. In general, there are three main causes determined the evapotranspiraiton, including radiation, aerodynamic and moisture conditions. Research shows that the wind speed presented a significant decreasing trend during the last 50 years in the study area^1^.In addition, the water consumption per unit area continued decline in the oasis area attribute to the improvement of irrigation technology. Thus, the decline of the aerodynamics condition, especially the decrease of wind speed, may offset the effect of temperature rise and further decreased the evapotranspiration^34^. And study also proved that the evaporation paradox phenomenon was a truth and the pan evaporation decreased during the last fifty years in Northwest China (including the Tarim Basin)^2^,^14^. So, the decrease of *OCI*_temperature_ may due to the decline of evapotranspiration in oases areas.

### Reconstruction of *OCI*_temperature_ and *T*_base_ data series

Because of the limitation in the spatial distribution of weather stations (there is only one weather station in the desert area, and its data collection started in 1999), we first calculated the OCI based on MODIS LST data from 2001 to 2013. Therefore, we needed to convert the *OCI*_LST_ to *OCI*_temperature_ to analyze the impacts of the oasis effect on the air temperature. Based on the regression relation between air temperature and LST (Fig. 3a,b), we calculated the *OCI*_temperature_ from 2001 to 2013. In addition, the *OCI*_temperature_ could be fitted by *R*_n_ in the oases. Therefore, we could easily obtain long time series data of *OCI*_temperature_ based on the *R*_n_ data from 1961 to 2000. Then, the *T*_base_ (air temperature without the oasis effect) sequence was reconstructed based on the following equation: *T*_base_ = *T*_observe_ + *OCI*_temperature_ (Fig. 3d). Our results showed that *T*_base_ exhibited a similar changing trend as *T*_observe_, and *T*_base_ was higher than *T*_observe_. This outcome proved that the oasis effect was a cooling effect.

**Figure 4:**
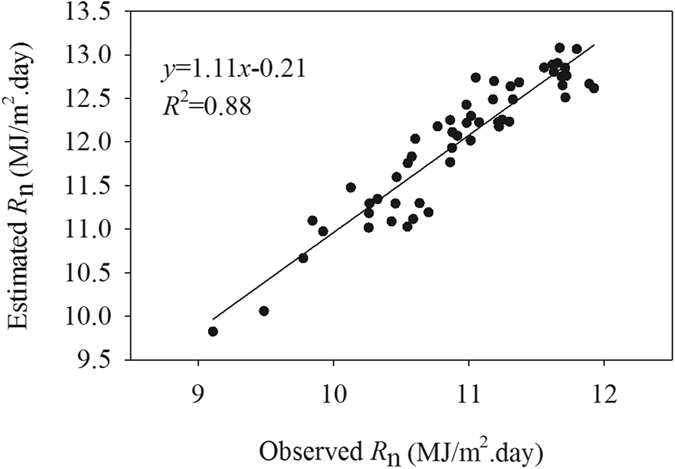
The scatter plot between estimated summer *R*_n_ and the observed summer *R*_n_ in three weather stations during 1993 to 2010. The estimated *R*_n_ was calculated by using Penman-Monteith equation, and the observed *R*_n_ collected from the only three radiation observation stations.

Considered there were only three weather stations can directly observe the radiation data (start from 1993) in the study area. Thus, we obtained the summer *R*_n_ by Penman-Monteith equation. In order to test the rationality of the estimated *R*_n_, the study analyzed the observe and estimated net radiation data of the three weather stations.Figure4 indicated that the estimated *R*_n_ has a significant linear correlation with the observed *R*_n_. Although, the estimated *R*_n_ usually higher 0.77–1.31 MJ/m^2^.day than the actual *R*_n_, the both all have the similar changing trend. Therefore, the estimated *R*_n_ can meet the requirement of the study.

### The reason for the temperature rise in the summers

Temperature increases is one of the most important heat-related issues in climate change research^22^,^35^,^36^. Studies have shown that the temperature rise is an objective fact in the entire Tarim Basin^20^,^37^. The main causes of the temperature rise have generally been attributed to atmospheric circulations, such as the El Niño-Southern Oscillation^20^, the Siberian High^22^, the North Atlantic Oscillation or the Arctic Oscillation^23^,^24^. However, the impacts of the oasis effect on temperature have long been ignored. In the Tarim Basin, the spatial distribution of weather stations is not uniform. There are a total of 43 weather stations in the basin; however, there is only one station in the desert and there are four stations distributed in the mountainous areas. The other 39 sites are distributed in the oasis areas, which account for only 11.5% of the total plains area. Thus, the observation data in these 39 sites could not reflect the real changing trend of temperature, especially in the summer.

To reveal the impacts of the oasis effect, we analyzed the changing trend of the *T*_base_, *T*_observe_ and *OCI*_temperature_ data series from 1961 to 2014 with the Mann-Kendall test. The test indicated that the *OCI*_temperature_ showed a significant decreasing trend, whereas the *T*_observe_ showed an highly significant increasing trend, which is consistent with previous research^38^. However, the trend test showed that the *T*_base_ only exhibit a slight increase trend which far less than the increase trend of *T*_observe_. The above results clearly indicate that the main reason for the summer temperature rise (*T*_observe_) was the decrease in *OCI*_temperature_. In addition, our research has proved that the oasis effect was clear in the warm season, and the OCI was the highest in the summer, followed by autumn and spring^15^. Therefore, the oasis effect should have similar impacts on the changing trend of spring and autumn air temperature.

**Figure 5:**
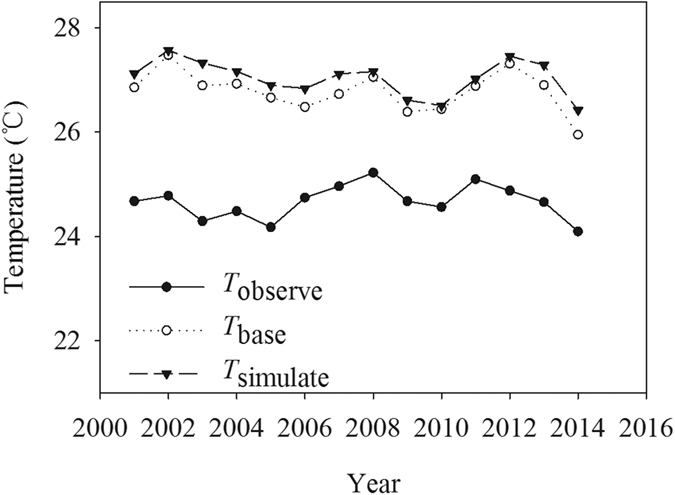
Average values of *T*_observe_, *T*_simulate_ and *T*_base_ in the entire Tarim Basin during summer from 2001 to 2014. The *T*_simulate_ in the desert and oases was calculated based on LST image data by the fitting formulas in Fig. 4a,b, respectively.

Besides the influence of the oasis effect, the arithmetic average value of air temperature based on the observed data could not entirely represent the temperature background of the study area. In this study, we obtained the average summer air temperature (*T*_simulate_) calculated at each raster based on the LST image data by using the fitting equation in Fig. 3a, b. Then, we calculated the arithmetic average summer air temperature (*T*_observe_) based on the observed data (Fig. 5). Indeed, the observed average temperature was 1.92–3.42 °C lower than the simulated temperature in the summer. Hence, the simple arithmetic average value of the summer temperature underestimated the real temperature background in the Tarim Basin.

Research showed that an enhanced warming in the semi-arid and arid area and will induce the expansion of dryland in the future^34^. Such warming trend also was detected in Tarim Basin based on the observed temperature data during the last 50 years^11^. However, the observed temperature (*T*_observe_) actually overestimated the warming trend in Tarim Basin due to the influence of oasis cold island and the uneven distribution of the weather stations (mostly distributed in oasis area). Our study showed that the temperature rise of *T*_base_ was 0.10 °C/decade, and the temperature rise of *T*_observe_ was 0.15 °C/decade. The significant decreasing trend of *OCI* was the main reason of the overestimate in warming trend. There are many factors can lead to the decrease of *OCI*, among which the decrease of evaporation may be one of the main reason^2^,^39^.

Based on our results, we believe that the influence of the oasis effect on temperature is significant, and the observed meteorological data not only underestimated the air temperature background but also overestimated the trend of air temperature rise, especially in the summer. The observed air temperature rise was the result of the decrease in the *OCI*_temperature_. In arid regions, the weather stations are generally distributed in the oases due to environmental limitations. Thus, the disturbance of the oasis effect on the observed air temperature is not unique to the Tarim Basin. Therefore, we must fully consider the oasis effect when studying the temperature change in arid regions.

## Conclusion

In Tarim Basin, oases had significant cold island effect, especially in summer. For a single oasis, the maximum *OCI*_LST_ and *OCI*_temperature_ were 3.82 °C and 2.67 °C, respectively. Although, the area of weak cold island was the main component of the cold island in all the oases, the area of the strong cold island had the largest influence on the *OCI*_LST_.

Based on surface energy balance equation, we concluded that the *R*_n_ can be as a key predictor of *OCI*_LST_ and *OCI*_temperature_. On the basis, this paper reconstructed the time series data of *OCI*_temperature_ in Tarim Basin from 1961 to 2000. And then, the paper obtained the long sequence of *T*_base_ during 1961 to 2014 by the equation (1). This study fond that the mean *T*_base_ of all the oases was 1.87, 2.00, 1.96, 1.88 and 1.85 °C higher than the *T*_observe_ in the 1960s, 1970s, 1980s, 1990s and since 2000, respectively.

The Mann-Kendall trend analysis indicated that the *T*_observe_ significantly increased during 1961–2014 (Test Z = 2.94, 99% confidence level), and the average temperature rise was 0.15 °C/decade. The *OCI*_temperature_ significantly decreased during the same period (Tset Z = −2.48, 95% confidence level), with an average decrease of 0.06 °C/decade. As to the *T*_base,_ it only showed a slight increase changing trend over the past 50 years(Tset Z = 1.94, 90% confidence level), and the average temperature rise only was 0.10 °C/decade, which far less than the increase trend of *T*_observe_. Based on above results, we believe that the observed air temperature rise was the result of the decrease in the *OCI*_temperature_. And the observed meteorological data not only underestimated the air temperature background but also overestimated the trend of air temperature rise, especially in the summer, due to the limitations in the spatial distribution of weather stations and the disturbance of oasis effect. In addition, the impacts of oasis effect on temperature should be an universal phenomenon in entire warm season of arid region (April to October in every year) based on our previous research^15^.

## Materials and Methods

### Study area

**Figure 6:**
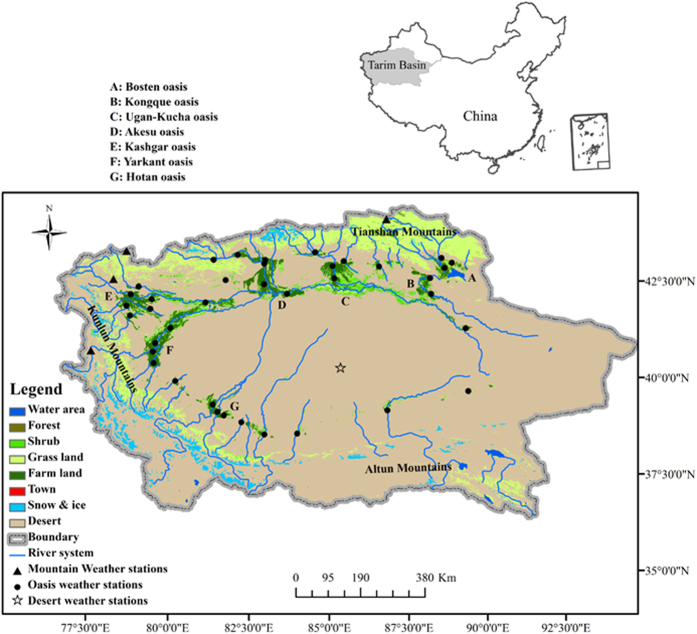
Sketch map of the study area. The map was created by the ArcGis software (Version 10.0). (http://www.esrichina.com.cn/softwareproduct/ArcGIS/).

The Tarim Basin is one of the largest closed basins in the world and is located in Xinjiang Province, Northwest China. This basin is surrounded by the Tianshan Mountains, the Kunlun Mountains and the Altun Mountains and is approximately 1500 km long from east to west and 600 km wide from north to south. The basin has an area of 53 × 10^4^ km^2^, and the altitude ranges from 800 m to 1300 m. The basin’s center is the famous Taklimakan Desert; therefore, the oases are only distributed along the river system at the edge of the basin. Based on the main river system, there are seven large oases, i.e., Bosten (around Bosten Lake), Kongque (along the Kongque River), Ugan-Kucha (along the Ugan-Kucha River), Akesu (along the Akesu River), Kashgar (along the Kashgar River), Yarkant (along the Yarkant River) and Hotan (along the Hotan River; Fig. 6). The Tarim Basin has a typical continental arid climate with scarce rainfall and high temperatures. In the plain area (mainly an oasis area), the annual temperature is approximately 11.7 °C and the precipitation is 76.2 mm. Cotton, wheat, rice and corn are the main crops in the oasis regions.

### Data collection

The MODIS land surface temperature (LST) data (MOD11A2 level data, from June to August of every year from 2001 to 2014) were downloaded from the website of the University of Maryland (https://ladsweb.nascom.nasa.gov/data/search.html). The temporal and spatial resolutions of the data are 8 days and 1 km x 1 km, respectively. The MODIS data were processed using the MODIS re-projection tool to generate a Tagged Image File Format in the WGS84 coordinate system. We used only pixels with LST error <1 K, as indicated by the quality assessment (QA) information included in the MOD11A2 dataset. In this paper, we converted the LST data from degrees Kelvin to degrees Celsius. In the MOD11A2 data set, band 1 and band 5 are LST in daytime and nighttime, respectively. In this study, we firstly calculated the average daily LST data based on the daytime and nighttime LST data. Then, the average LST in the summer (from June to August, including the LST data in Julian days of 153,161,…241) of every year from 2001 to 2014 were calculated using ArcGIS software.

The LUCC data (MOD12Q1 from 2001 to 2013, land cover at 500 m resolution) were also downloaded from the University of Maryland (https://ladsweb.nascom.nasa.gov/data/search.html). The data were first processed by mosaic, band match and re-projection methods and then resampled (using the nearest neighbor method) to 1 km resolution. In this study, we adopted the global vegetation classification system of International Geosphere-Biosphere Program (IGBP) for the LUCC data.

The meteorological data, such as daily mean, max and min temperature, relative air humidity and max sunshine hours, among others, were collected from 43 weather stations from 1961 to 2014. In addition, we downloaded the digital elevation model (DEM) data from the website of the U.S. Geological Survey (*USGS*; http://tahoe.usgs.gov/DEM.html, data with a 30 m grid). Based on the DEM data, we defined the mountainous areas (elevation higher than 1500 m) and the plains areas (elevation lower than 1500 m).

### Analysis methods

In the plains area of the Tarim Basin, we defined the water bodies, croplands, cropland-natural vegetation mosaic, urban areas, permanent wetlands and the natural vegetations (including deciduous broadleaf forest, mixed forest, colosed shrublands, open shrublands, woody savannas, savannas and grasslands) as oases and extracted the oasis borders from 2001 to 2013 based on the LUCC data and the administrative boundary of each oasis (including Bosten, Konque, Ugan-Kucha, Akesu, Kashgar, Yarkant and Hotan oasis). On the basis, the study considered the boundary of each oasis as the benchmark, and then outward buffered 50 km and took the outward buffer zone as the surrounding desert zone of each oasis. We excluded the mountainous and other oases area from the buffer zone analysis. Furthermore, we obtained the summer LST data of oases and the surrounding desert areas from 2001 to 2013 by using ArcGIS software based on the above boundary files.

In this study, The *OCI* was defined as the difference between the average LST (or air temperature) in the desert and the oasis area^27^, and all OCI was calculated in summer season. The daily *R*_n_ (net radiation) was calculated using the Penman-Monteith equation^40^. All daily meteorological data, including temperature and *R*n were calculated to obtain a monthly average, which was finally calculated to obtain the seasonal data in every year for each weather station. In addition, the mean summer air temperature and the net radiation of each oasis from 2001 to 2013 were calculated based on the oasis boundary and weather station data. All these boundary and LST data were obtained by using ArcGis software. The changing trend of the time series data was analyzed with the Mann-Kendall method^41^.

## Additional Information

**How to cite this article**: Hao, X. *et al.* The oasis effect and summer temperature rise in arid regions - case study in Tarim Basin. *Sci. Rep.* **6**, 35418; doi: 10.1038/srep35418 (2016).
